# Nursing for conical telescopic crown prosthodontics in treating periodontitis accompanied by dentition defects

**DOI:** 10.4314/ahs.v24i4.47

**Published:** 2024-12

**Authors:** Lina Hu, Miao Liang, Feng Zhu, Rongrong Nie

**Affiliations:** Nanjing Stomatological Hospital, Medical School of Nanjing University, 30 Zhongyang Road, Nanjing 210000, Jiangsu Province, China

**Keywords:** Dentition, defect, nursing, periodontitis, prosthodontics

## Abstract

**Objective:**

We aimed to investigate the nursing technique and outcomes of conical telescopic crown prosthodontics for patients with periodontitis accompanied by dental defects.

**Methods:**

One hundred patients with chronic periodontitis and dental problems from January 2018 to August 2020 were enrolled. They were randomly assigned to an observation group (n=50) and a control group (n=50). The control group received traditional nursing, whereas the observation group received comprehensive nursing. Comparisons were made between the indices related to chewing function and periodontal condition, inflammatory factors, quality-of-life score, and nursing satisfaction rate.

**Results:**

The masticatory efficiency and absorbance were greater in the observation group than in the control group following nursing (P<0.05). The observation group had lower levels of plaque index, debris index, sulcus bleeding index, and periodontal probing depth, as well as levels of C-reactive protein, tumor necrosis factor-alpha, and interleukin-6 (P<0.05). The quality-of-life score of the observation group was significantly higher than that of the control group (P<0.05). The nursing satisfaction rate of the observation group was significantly higher than that of the control group (P<0.05).

**Conclusion:**

Comprehensive nursing for conical telescopic crown prosthodontics can improve the chewing function and periodontal conditions of patients with periodontitis accompanied by dentition defects.

## Introduction

Chronic periodontitis is the infection of the gums caused by local factors. In the initial phase, inflammation accumulates and gradually aggravates, extending from the gums to the periodontal membrane and even the cementum. This leads to the development of dental calculus, periodontal biofilms, and soft deposits. The smoothness of the tooth root surface is compromised over time.^1^-^3^ Finally, it results in reduced chewing ability, difficulty in eating, and poorer quality of life.^4^-^6^

Prosthodontics is the main method for treating dental diseases.^7^ It is also the major technique for clinically treating dentition defects. Fixed or removable dentures are mainly used to repair the integrity of the dentition, thereby helping patients restore normal physiological function and oral aesthetics and improving the masticatory efficiency.^8^,^9^ Lately, the use of a conical telescopic crown in this treatment has become more common, but it poses various risks and may impact theffectiveness of prosthodontics.^10^ Therefore, reasonable nursing measures are necessary.

Thus, the current study investigated the nursing technique and outcomes of conical telescopic crown prosthodontics for patients with periodontitis accompanied by dental defects, aiming to provide valuable evidence for clinical practice.

## Materials and methods

### General data

We enrolled and divided 100 patients with chronic periodontitis and dentition defects from January 2018 to August 2020 into an observation group (n=50) and a control group (n=50) using a random number table. The control group consisted of 24 males and 26 females aged from 20 to 57 years, with an average age of (38.45±9.26) years. The observation group included 23 males and 27 females aged 20 to 58 years, with a mean age of (38.69±9.17) years. The two groups were comparable in terms of age and sex without statistically significant differences (P>0.05). The ethics committee of the hospital reviewed and approved the current study, with the patients giving their informed consent.

### Inclusion and exclusion criteria

The following criteria were utilized for inclusion: 1) patients diagnosed with chronic periodontitis and dentition defects after an oral examination, 2) those aged ≥20 and <60 years old, and 3) those with clear consciousness and willingness to participate in the study.

The patients who had mental disorders or disturbances of consciousness, concomitant severe systemic infection, blood system disease or coagulation disorder, or heart, lung, liver, or kidney dysfunction were excluded.

### Methods

Traditional nursing was carried out for the control group. The doctors provided the patients with information about the processes and safety measures involved in conical telescopic crown prosthodontics, and the patients actively collaborated with the doctors to undergo the entire treatment.

Comprehensive nursing was performed for the observation group as follows: *1) Cognitive intervention:* Once the treatment plan was determined, the patients were provided with comprehensive information regarding conical telescopic crown prosthodontics, including the steps, duration, and associated expenses. Subsequently, they were allowed to ask any questions. Their questions, if any, were patiently addressed, and they were provided with information regarding post-treatment precautions.

*2) Mental nursing:* The medical staff communicated with the patients and listened carefully to them while understanding their psychological status. Furthermore, the origins of adverse feelings were examined, based on which solace and guidance were provided. After wearing the telescopic crown denture, the patient needed to be comforted to be adaptable.

*3) Oral nursing:* The patients gargled using suitable mouthwash daily after meals. During gargling, the solution needed to be fully stirred, and the oral cavity was rinsed from the left and right sides, the upper and lower sides, and the anterior and posterior direction of the tongue. Besides, the denture was carefully cleansed to avoid leaving food residues.

### Evaluation of chewing function indices

The chewing function indices included the masticatory efficiency and absorbance in the mastication test. The masticatory efficiency was determined by measuring the weight of both chewed food and food residues. The formula for calculating masticatory efficiency: (total weight of chewed food - weight of food residues)/total weight of chewed food×100%. The measurement of absorbance was conducted at a wavelength of 590 nm.

### Evaluation of periodontal condition indices

The indices related to periodontal condition included plaque index, debris index, sulcus bleeding index, and the depth of periodontal probing. The sulcus bleeding index was determined as follows: The periodontal probe was first inserted 1 mm below the lower sulcus margin to slide, and bleeding was observed. The bleeding severity determined the score, which ranged from 0 to 5 points. The levels of plaque and debris were assessed using the 4-point Likert scale, ranging from 0 to 3 points. A higher score indicated more periodontal plaques and debris. Furthermore, the depth of the periodontal pocket was assessed using the periodontal probe.

### Detection of inflammatory factors

The inflammatory factors included C-reactive protein (CRP), tumor necrosis factor-alpha (TNF-α), and interleukin-6 (IL-6). The immunity transmission turbidity method was employed to test CRP, whereas TNF-α and IL-6 were detected through enzyme-linked immunosorbent assay.

### Assessment of quality of life

The assessment of the quality of life was conducted by employing the Brief Version of the World Health Organization Quality of Life Scale.^11^ This scale comprises physical health, psychological health, social relationships, and environmental health, with each item being assigned a score between 0 and 100 points. A higher score indicated a better quality of life.

### Assessment of nursing satisfaction

Nursing satisfaction was surveyed using a self-made questionnaire (the validity was 0.90 and the confidence was 0.88) with a maximum score of 100 points. In the questionnaire, the cut-off values were 60 and 80 points, and <60 points indicated being dissatisfied, 60-80 points being moderately satisfied, and >80 points being very satisfied. Rate of overall satisfaction = very satisfied rate + moderately satisfied rate.

### Statistical analysis

SPSS 22.0 software (IBM Inc., USA) was used for statistical analysis. The χ2 test was used to analyze the count data (n), while t test was used for the measurement data (mean ± standard deviation). The difference was considered statistically significant, with a P-value lower than 0.05.

## Results

### Chewing function indices

Both groups exhibited a significant increase in the masticatory efficiency and absorbance in the masticatory test after nursing (P<0.05). Additionally, the observation group had higher masticatory efficiency and absorbance than those of the control group (P<0.05) (Table 1).

**Table 1:** Chewing function indices ()

Group	Time	Masticatory efficiency	Absorbance in the mastication test
Control group (n=50)	Before nursing	56.34±8.51	0.352±0.059
	After nursing	72.17±10.57^#^	0.421±0.064^#^
Observation group (n=50)	Before nursing	56.67±8.40	0.355±0.058
	After nursing	84.94±n.36^#^*	0.483±0.062^#^*

# P<0.05 *vs.* before nursing in the same group

* P<0.05 *vs.* Control group

### Periodontal condition indices

The plaque index, debris index, sulcus bleeding index, and periodontal probing depth significantly decreased in both groups after nursing (P<0.05). Furthermore, these indices were lower in the observation group than in the control group (P<0.05) (Table 2).

**Table 2:** Periodontal condition indices ()

Group	Time	Plaque index (point)	Debris index (point)	Sulcus bleeding index (point)	Periodontal probing depth (mm)
Control group (n=50)	Before nursing	2.03±0.52	2.11±0.56	3.89±1.02	6.75±1.42
	After nursing	1.51±0.40^#^	1.57±0.43^#^	2.73±0.87^#^	5.21±1.13^#^
Observation group (n=50)	Before nursing	2.01±0.55	2.08±0.58	3.81±1.15	6.68±1.45
	After nursing	1.14±0.36^#^*	1.17±0.34^#^*	1.96±0.64^#^*	4.07±1.06^#^*

# P<0.05 *vs.* before nursing in the same group

* P<0.05 *vs.* Control group

### Inflammatory factors

The levels of CRP, TNF-α, and IL-6 decreased in both groups after nursing (P<0.05). In addition, the observation group had lower levels of CRP, TNF-α, and IL-6 than those of the control group (P<0.05) (Table 3).

**Table 3:** Inflammatory factors ()

Group	Time	CRP (mg/L)	TNF-α (mg/L)	IL-6 (ng/L)
Control group (n=50)	Before nursing	9.83±2.35	16.95±3.30	26.41±3.59
	After nursing	7.20±1.87^#^	13.36±2.61^#^	22.78±2.17^#^
Observation group (n=50)	Before nursing	9.71±2.40	16.80±3.42	26.28±3.61
	After nursing	5.38±1.49^#^*	10.47i1.85^#^*	20.55±1.72^#^*

# P<0.05 *vs.* before nursing in the same group

* P<0.05 *vs.* Control group

### Scores for the quality of life

The quality-of-life scores of both groups exhibited an increase after nursing (P<0.05). Additionally, the observation group had a higher score than that of the control group (P<0.05) (Table 4).

**Table 4:** Quality-of-life scores (point)

Group	Time	Physical health	Psychological health	Environmental health	Social relationship
Control group (n=50)	Before nursing	70.81±5.20	70.23±5.14	70.34±4.91	70.58±5.09
	After nursing	77.34±6.42^#^	77.87±6.17^#^	76.46±5.23^#^	76.73±5.40^#^
Observation group (n=50)	Before nursing	70.96±5.17	70.45±5.13	70.48±4.99	70.20±5.16
	After nursing	84.05±6.59^#^*	84.39±6.28^#^*	82.47±5.38^#^*	83.62±5.71^#^*

# P<0.05 *vs.* before nursing in the same group

* P<0.05 *vs.* control group

### Rate of satisfaction with nursing

The nursing satisfaction rate of the observation group was significantly higher than that of the control group (98.00% vs. 86.00%, P<0.05) (Table 5).

**Table 5:** Nursing satisfaction rate [n (%)]

Group	n	Very satisfied	Moderately satisfied	Dissatisfied	Overall satisfaction rate
Control group	50	21 (42.00%)	22 (44.00%)	7 (14.00%)	43 (86.00%)
Observation group	50	26 (52.00%)	23 (46.00%)	1 (2.00%)	49 (98.00%)*

* P<0.05 *vs.* Control group

## Discussion

Dentition defect, which is frequently seen in individuals with chronic periodontitis, refers to the harm caused to the structure of teeth. This not only affects the appearance of teeth but also significantly hampers the ability to chew, resulting in difficulties with eating on a daily basis.^12^-^14^ Consequently, timely treatment is necessary for patients with periodontitis accompanied by dentition defects.^15^ In recent years, the conical telescopic crown has been commonly used for prosthodontic treatment owing to high stability and preservation of periodontal and subbase tissues.^16^ During treatment, however, some patients tend to have emotions of fear of the lack of understanding of conical telescopic crown prosthodontics, so proper nursinmeasures are required.^17^

Conventional nursing measures are dominated by simple guidance, whereas not enough attention is paid to other nursing issues (such as unhealthy emotions), so the nursing effect is far from satisfactory. In contrast, comprehensive nursing focuses on details and advocates for optimizing all aspects of nursing, thereby achieving the goal of seamless nursing and improving the quality of service.^18^ In this study, the masticatory efficiency and absorbance were greater in the observation group than in the control group following nursing. Moreover, the observation group had reduced plaque index, debris index, sulcus bleeding index, periodontal probing depth, as well as levels of CRP, TNF-α, and IL-6 than those of the control group. Probably, incorporating cognitive intervention and mental nursing into the comprehensive nursing plan not only improved the patients' comprehension of conical telescopic crown prosthodontics but also helped them willingly participate in the treatment.^19^ Additionally, it enhanced the patients' awareness of oral health, leading to their active maintenance of oral hygiene and improvement of periodontal cleanliness.^20^ Furthermore, the observation group had a higher quality-of-life score than that of the control group, also with a higher overall satisfaction rate. Possibly, comprehensive nursing enhanced the patients' chewing ability and minimized the negative impact of masticatory dysfunction on their quality of life, ultimately increasing their satisfaction degree.^21^

## Conclusion

To summarize, providing comprehensive nursing during the use of conical telescopic crown prosthodontics can enhance the ability to chew and periodontal health, relieve inflammation in the gums, and ultimately improve the quality of life and satisfaction of the patients with periodontitis accompanied by dental defects.
